# Adherence to anti-vectorial prevention measures among travellers with chikungunya and malaria returning to Australia: comparative epidemiology

**DOI:** 10.1186/s13104-018-3695-9

**Published:** 2018-08-14

**Authors:** Dillon Charles Adam, Chau Minh Bui, Anita Elizabeth Heywood, Mohana Kunasekaran, Mohamud Sheikh, Padmanesan Narasimhan, Chandini Raina MacIntyre

**Affiliations:** 1 Kirby Institute, University of New South Wales (UNSW) Sydney, Sydney, Australia; 2 School of Public Health & Community Medicine, University of New South Wales (UNSW) Sydney, Sydney, Australia; 3 College of Public Service & Community Solutions, Arizona State University (ASU), Tempe, AZ USA

**Keywords:** Communicable diseases, Travel medicine, Health behavior, Chikungunya, Malaria

## Abstract

**Objective:**

Compare the adoption and adherence to health protection behaviours prior to and during travel among international Australian travellers who return to Australia with notified chikungunya or malaria infection. This information could inform targeted health promotion and intervention strategies to limit the establishment of these diseases within Australia.

**Results:**

Seeking travel advice prior to departure was moderate (46%, N = 21/46) yet compliance with a range of recommended anti-vectorial prevention measures was low among both chikungunya and malaria infected groups (16%, N = 7/45). Reasons for not seeking advice between groups was similar and included ‘previous overseas travel with no problems’ (45%, N = 9/20) and ‘no perceived risk of disease’ (20%, N = 4/20). Most chikungunya cases (65%, N = 13/20) travelled to Indonesia and a further 25% (N = 5/20) visited India, however most malaria cases (62%, N = 16/26) travelled to continental Africa with only 12% (N = 3/26) travelling to India. The majority (50%, N = 10/20) of chikungunya cases reported ‘holiday’ as their primary purpose of travel, compared to malaria cases who most frequently reported travel to visit friends and family (VFR; 42%, N = 11/26). These results provide import data that may be used to support distinct public health promotion and intervention strategies of two important vector-borne infectious diseases of concern for Australia.

**Electronic supplementary material:**

The online version of this article (10.1186/s13104-018-3695-9) contains supplementary material, which is available to authorized users.

## Introduction

Global travel is responsible for the importation of many vector-borne diseases around the world including chikungunya and malaria, however neither is endemic in Australia [1, 2]. As the volume of international return departures from Australia to malaria and chikungunya endemic countries increases, so does the risk of importation and subsequent establishment. Seeking pre-travel health advice and adherence to AVPM has been shown to reduce the risk of individual infection from vector-borne diseases whilst travelling [3–5]. As chikungunya and malaria are two important yet distinct vector-borne diseases of public health significance, we aimed to understand the travel characteristics and behaviours of those who become infected with these diseases whilst overseas. These data could inform future public health interventions and reduce the risk of endemic establishment within Australian borders. We have previously reported on the broad demographics and characteristics of travellers who acquired malaria and chikungunya among a range of other notifiable diseases associated with travel including typhoid, paratyphoid, measles, hepatitis A, hepatitis E [6]. However, detailed information including health behaviours during and prior to travel such as the adoption of recommended AVPM has yet to be reported.

## Main text

### Methods

Confirmed cases of chikungunya and malaria were defined in accordance with national definitions [7]. Cases notified to Departments of Health in the states of New South Wales (NSW) and Victoria (VIC) from February 2013 to January 2014 were identified, and permission to contact each case was acquired. Verbal informed consent was reaffirmed by participants prior to administering the questionnaire via telephone. Eligible participants were Australian resident travellers (including temporary residents) who returned from overseas with malaria or chikungunya notified to health authorities in NSW or VIC during the study period. Temporary visitors, and all arriving migrants (including refugees) were excluded. The questionnaire collected details on case demographics, pre-travel health seeking behaviours, characteristics of travel and the use of AVPM whilst travelling such as insect repellent, sleeping in accommodation with air conditioning or bed nets, and covering arms and legs. Reasons for not seeking pre-travel health advice was also obtained. The full methods employed and questionnaire used are described comprehensively in our previous study from which this dataset was obtained [6].

Descriptive and statistical analysis was completed using IBM SPSS Statistics for Windows (v22.0). Distributions of variables were presented as percentages. Continuous variables were tested for significance between chikungunya and malaria groups using independent t-tests and categorical variables compared using both Chi square and fisher exact tests depending on response rates.

### Results

During the study period, a total of 182 malaria cases and 55 chikungunya cases were notified to state authorities in NSW and VIC. Twenty-six malaria cases (14.3%) and 20 chikungunya cases (36%) gave consent to participate in the enhanced surveillance survey. The mean age for all respondents was 44 years. The mean age of chikungunya cases was higher than malaria cases (54 versus 37 years, P < 0.0001), respectively. Sixty-five percent (N = 13/20) of chikungunya cases were female compared to 23% (N = 6/26) of malaria cases (P = 0.05). The migrant status (born in Australia versus overseas) of cases between groups was not significantly different although among migrant chikungunya cases, however average time since migrating to Australia was 24.4 years for chikungunya cases compared to 9.5 years for malaria cases (P < 0.001). Additional demographics are available in Additional file 1: Table S1.

### Characteristics of travel

The median trip length for all cases completing the survey was 30 days and ranged from 6 to 304 days. The mean trip length for chikungunya cases was lower than malaria cases with 16.5 days and 47.5 days, respectively (P = 0.04). Eighty-one percent (N = 20/26) of malaria cases travelled for greater than 1 month compared to 70% (N = 14/20) of chikungunya cases who travelled less than 1 month. VFR was the most commonly reported purpose of travel (41%, N = 19/46) followed by ‘holiday’ (30% N = 14/46) and ‘business’ (22%, N = 10/46). Among malaria cases, 62% (N = 16/26) of trips were to continental Africa, followed by Asia (27%, N = 7/26) and Oceania (11%, N = 3/26). Ghana, India and Nigeria were the most visited countries, with four malaria cases reporting travel to each of these countries. Among chikungunya cases, Indonesia was the most commonly reported travel destination (65%, N = 13/20), followed by India (25%, N = 5/20) and the Philippines (10%, N = 2/20). Table 1 describes additional travel characteristics of cases who completed the questionnaire stratified by disease.

**Table 1:** Travel characteristics of malaria and chikungunya cases completing the enhanced survey

	Total	Malaria		Chikungunya		P value^†^
	Number	Number	Percent (%)	Number	Percent (%)	
Trip length						
1 to < 2 weeks	9	1	4	8	42	0.005
2 weeks to < 1 month	10	4	15	6	32	
1 to < 2 months	13	10	38	3	16	
2 to < 3 months	3	2	8	1	5	
3+ months	10	9	35	1	5	
Reason for travel						
Holiday	14	4	15	10	50	0.06
Business/conference	10	8	31	2	10	
VFR	19	11	42	8	40	
Study	1	1	4	0	0	
Other	2	2	8	0	0	
Trip booking method						
Internet	12	8	32	4	21	0.1
Travel agent	22	14	56	8	42	
Other	10	3	12	7	37	
Time from booking to departure (months)						
<1	15	11	44	4	21	0.5
1–3	17	9	36	8	42	
>3	12	5	20	7	37	
Five-year travel frequency						
1–2	10	8	31	2	11	0.02
3–5	22	8	31	14	74	
> 5	13	10	38	3	16	

^†^ P value for differences in proportional responses between malaria and chikungunya groups using Chi square test

### Health behaviours

Table 2 describes pre-travel health seeking behaviours and AVPM employed by chikungunya and malaria cases responding to the enhanced survey. Fifty-four percent (N = 25/46) of all cases did not seek advice prior travel. Seeking pre-travel advice was not significantly different between groups (P = 0.06). Forty-five percent of cases (N = 9/20) said ‘previous overseas travel with no problems’ was the reason for not seeking advice however only 20 cases overall responded. Most survey respondents used at least one AVPM variably (sometimes, occasionally or always) except one chikungunya case (98%, N = 44/45). Only seven subjects used all preventative measures surveyed though compliance (sometimes, occasionally or always) differed between them. Specific to malaria, around half (52%, N = 13/25) reported not using chemoprophylaxis, and of those who took medications, 58% (N = 7/12) did not comply with the recommended course. Open field qualitative analysis (not shown) showed heat concerns among both chikungunya and malaria cases was the most frequently cited reason (68%, N = 5/8) for disuse of AVPM such as using nets and wearing long sleeve shirts and pants. Additional quantitative results of travel health behaviours can be seen in Table 2.

**Table 2:** Travel health seeking behaviour and AVPM of malaria and chikungunya cases in Australia

	Total	Malaria		Chikungunya		P value^†^
	Number	Number	Percent (%)	Number	Percent (%)	
Sought pre-travel advice						
No	25	11	42	14	70	0.06
Yes	21	15	58	6	30	
Reasons for not seeking advice						
No perceived risk of diseases	4	1	11	3	27	0.3
Concerns for medication side effects	1	1	11	0	0	
Time constraints	3	2	22	1	9	
Travelled overseas previously with no problems	9	5	56	4	36	
Previous travel to country of origin with no problems	3	0	0	3	27	
Frequency of insect repellent use						
Never	21	12	46	9	47	0.2
Occasionally	8	5	19	3	16	
Often	4	4	15	0	0	
Always	12	5	19	7	37	
Time of insect repellent use						
Night only	17	10	77	7	70	0.7
Day and night	6	3	23	3	30	
Frequency of wearing long sleeve shirts/pants						
Never	10	4	15	6	32	0.2
Occasionally	16	8	31	8	42	
Often	7	6	23	1	5	
Always	12	8	34	4	21	
Frequency of mosquito net use						
Never	33	17	65	16	84	0.3
Occasionally	4	2	8	2	11	
Often	2	2	8	0	0	
Always	6	5	19	1	5	
Frequency of air-conditioning or screened accommodation						
Never	10	8	31	2	11	0.4
Occasionally	7	3	12	4	21	
Often	3	2	8	1	5	
Always	25	13	50	12	63	

^†^ P value for differences in proportional responses between malaria and chikungunya groups using Chi square test

### Discussion

In this study we show important differences between the characteristics and behaviours of travellers returning to Australia with chikungunya and malaria. Travel for VFR purposes remains an important risk group for vector-borne diseases, particularly for malaria however less so for chikungunya, who more often reported holiday as their primary purpose of travel (Table 1). VFR travellers are recognised as an important risk group in Australia due in part to a lower perceived risk of infection when returning to their country of birth or parent’s country of birth along with an increasing migrant resident population [8–11]. Both immigrant and Australian-born travellers with migrant parents departing to continental Africa or India for longer than 1 month appear at risk of malaria infection and importation. In comparison, all travellers regardless of purpose or migrant status who travel to Asia, especially Indonesia, might be considered at risk for chikungunya infection and importation, even on short trips. This is likely to be reflective of the number of Australian holiday travellers to Indonesia and does not necessarily mean that Indonesia is a higher risk destination.

We also show that adherence to the full-range of recommend AVPM among cases of chikungunya and malaria was equally low. AVPM such as the use of mosquito repellent, mosquito nets, staying in air-conditioned and screened accommodation and covering arms and legs form a well-established range of complimentary strategies designed to prevent vector-borne infections such as chikungunya and malaria [12]. The use of mosquito repellent was particularly under-utilised, with nearly half of all cases reporting never using repellent. Furthermore, there were apparent misconceptions about the correct use of mosquito repellent among chikungunya cases. The most significant chikungunya competent vectors are principally daylight biting mosquitoes (between dawn and dusk) [12], however, 70% of chikungunya cases who used repellent reported only applying so at night, perhaps indicating a higher awareness of the night-time biting patterns of anopheline mosquitos, the vector for malaria. This may be the result of malaria specific health messaging which focuses on dusk/dawn biting risk but is not applicable to chikungunya and other mosquito-borne diseases [13].

These data have implications for targeted public health promotion and disease prevention activities. For example, interventions promoting under-utilised AVPM could be designed to specifically target different traveller profiles, such as those who travel to Africa or Indonesia and become infected with malaria and chikungunya, respectively. For those travelling to Indonesia, interventions targeting the apparent low awareness of daylight biting mosquitos may improve and correct repellent-use therefore reducing the risk of infection from chikungunya. The sporadic and incorrect use of most AVPM across both groups may be due to the limited uptake of pre-travel health seeking behaviours reported among Australasian travellers and low disease risk perceptions based on prior incident free travel experiences [14–16]. This is consistent with the results of our survey (Table 2). Seeking pre-travel health advice has been shown to increase compliance to AVPM among travellers by improving awareness of vector-borne infectious diseases and correcting inaccurate risk perceptions [3–5]. As 70% chikungunya cases reported they did not seek pre-travel health advice compared to 42% of malaria cases, this may also provide an opportunity for targeted interventions based on traveller profiles, such as holiday travellers vs. travellers VFR.

Chikungunya and malaria are both important vector-borne infectious diseases of concern for Australian travellers. There appears a need for nuanced health promotion interventions targeting low awareness and inaccurate risk perceptions among these travellers. This includes educating travellers on the biting patterns of mosquito vectors of chikungunya and malaria. The success of such intervention strategies may reduce the individual risk of disease and subsequently the potential for chikungunya and malaria establishment within Australian borders as ongoing biosecurity concerns of domestic vector establishment continues [17–19]. Additional research on the efficacy and impact of AVPM interventions designed to increase overall awareness of AVPM among travellers departing Australia is needed. Ongoing enhanced surveillance will be an important tool to understand risk factors and evaluate the impact of interventions and strategies in the future.

## Limitations

The main limitation of this study was the small sample size of notified cases who consented to the enhanced surveillance survey: 14.3% (N = 26/186) of malaria cases and 36% (N = 20/55) of chikungunya cases. The reliance on obtaining permission from the case’s treating doctor to contact each case likely limited recruitment. We also cannot know if there was any significant difference in case behaviour between those who gave consent and those who did not which limits interpretation of the results. Even so, some significant differences were identified. Additionally, the study relied inherently on retrospective self-reported data meaning the results may be subject to recall bias. This study was also limited to cases notified in NSW and VIC. While these are the most populous states in Australia, no data was collected from Queensland (QLD), where, due to climate factors the threat of establishment is present means the results may not be generalizable. Extending future studies into Queensland where greater chikungunya and malaria receptive zones are present and increasing sample size would provide a more generalizable dataset and opportunities for comparison of travel behaviours between states. Despite this, the information collected here is valuable as it is not routinely collected by Australia’s National Notifiable Disease Surveillance System (NNDSS). We recommend the inclusion of relevant epidemiological questions such as travel behaviours additional to the currently mandated case reports submitted to the NNDSS for malaria and chikungunya. This would provide important and accurate data on these cases to the benefit of future surveillance and public health intervention strategies. As the number of cases of chikungunya and malaria cases returning to Australia each year is relatively small, any burden would likely be minimal.

## Additional file

**Additional file 1: Table S1.** Demographics of malaria and chikungunya cases completing the enhanced survey (n = 46), Feb 2013–Jan 2014.

## Abbreviations

AVPM: anti-vectorial prevention measures
NNDSS: National Notifiable Disease Surveillance System
NSW: New South Wales
QLD: Queensland
VIC: Victoria
VFR: visiting friends and relatives
