# Supplementary material for: Adherence to anti-vectorial prevention measures among travellers with chikungunya and malaria returning to Australia: comparative epidemiology
Source: BMC Res Notes. 2018 Aug 14;11:590. doi: 10.1186/s13104-018-3695-9 (PMC6092863; doi:10.1186/s13104-018-3695-9)
Supplement: Supplementary file 1 — Additional file 1: Table S1. Demographics of malaria and chikungunya cases completing the enhanced survey (n = 46), Feb 2013–Jan 2014. [file 13104_2018_3695_MOESM1_ESM.docx]

| Additional file 1: Table S1. Demographics of malaria and chikungunya cases completing the enhanced survey (n=46), Feb 2013- Jan 2014 | | | | | | |
| --- | --- | --- | --- | --- | --- | --- |
|  | Total | Malaria | | Chikungunya | | P value^†^ |
|  | Number | Number | Percent | Number | Percent |  |
| **Sex** |  |  |  |  |  |  |
| Male | 27 | 20 | 77% | 7 | 35% | 0.004 |
| Female | 19 | 6 | 23% | 13 | 65% |  |
| **Age (years)** |  |  |  |  |  |  |
| 0-19 | 2 | 2 | 8% | 0 | 0% | 0.05 |
| 20-39 | 16 | 14 | 54% | 2 | 10% |  |
| 40-59 | 20 | 8 | 31% | 12 | 60% |  |
| >60 | 8 | 2 | 8% | 6 | 30% |  |
| **Region of Birth** |  |  |  |  |  |  |
| Australia | 20 | 8 | 31% | 12 | 60% | 0.03 |
| South and Central Asia | 7 | 3 | 12% | 4 | 20% |  |
| South-East Asia | 3 | 1 | 4% | 2 | 10% |  |
| Pacific (including New Zealand) | 3 | 3 | 12% | 0 | 0% |  |
| Sub-Saharan Africa | 9 | 9 | 35% | 0 | 0% |  |
| Europe | 4 | 2 | 8% | 2 | 10% |  |
| **Migrant status of cases** |  |  |  |  |  |  |
| Australian born, Australian born parents | 15 | 5 | 19% | 10 | 50% | 0.08 |
| Australian born, migrant parents | 5 | 3 | 12% | 2 | 10% |  |
| Migrant | 26 | 18 | 69% | 8 | 40% |  |
| **Education** |  |  |  |  |  |  |
| Graduate Degree | 7 | 3 | 12% | 4 | 21% | 0.6 |
| Undergraduate Degree | 15 | 9 | 36% | 6 | 32% |  |
| Trade certificate | 8 | 5 | 20% | 3 | 15% |  |
| Secondary School | 11 | 5 | 20% | 6 | 32% |  |
| Primary School | 1 | 1 | 4% | 0 | 0% |  |
| **Migrant years (mean)** | 16.3 | 9.5 |  | 24.4 |  | 0.001 |

^†^ P Value for differences in proportional responses between malaria and chikungunya groups using chi-square test
